# Energetic benefits of resting behaviour in humpback whale mother–calf pairs revealed by biologging and UAS-photogrammetry

**DOI:** 10.1093/conphys/coag041

**Published:** 2026-07-07

**Authors:** Augusta Hollers, William T Gough, Martin van Aswegen, Andrew Szabo, Lewis Evans, Jens J Currie, Ashley M Blawas, Andreas Fahlman, Jeremy A Goldbogen, Lars Bejder

**Affiliations:** Marine Mammal Research Program, Hawaiʻi Institute of Marine Biology, University of Hawaiʻi at Mānoa, 46-007 Lilipuna Road, Kaneohe, HI 96744, USA; Marine Mammal Research Program, Hawaiʻi Institute of Marine Biology, University of Hawaiʻi at Mānoa, 46-007 Lilipuna Road, Kaneohe, HI 96744, USA; Hopkins Marine Station, Stanford University, 120 Ocean View Blvd, Pacific Grove, CA 93950, USA; Marine Mammal Research Program, Hawaiʻi Institute of Marine Biology, University of Hawaiʻi at Mānoa, 46-007 Lilipuna Road, Kaneohe, HI 96744, USA; Marine Mammal Research Program, Hawaiʻi Institute of Marine Biology, University of Hawaiʻi at Mānoa, 46-007 Lilipuna Road, Kaneohe, HI 96744, USA; Alaska Whale Foundation, PO Box 1927, Petersburg, AK 99833, USA; Marine Mammal Research Program, Hawaiʻi Institute of Marine Biology, University of Hawaiʻi at Mānoa, 46-007 Lilipuna Road, Kaneohe, HI 96744, USA; Marine Mammal Research Program, Hawaiʻi Institute of Marine Biology, University of Hawaiʻi at Mānoa, 46-007 Lilipuna Road, Kaneohe, HI 96744, USA; Pacific Whale Foundation, 300 Ma’alaea Road, Suite 211, Wailuku, HI 96793, USA; Hopkins Marine Station, Stanford University, 120 Ocean View Blvd, Pacific Grove, CA 93950, USA; Fundación Oceanogràfic, Carrer d’Eduardo Primo Yúfera, 1B, 46013 València, Spain; IFM, Linköping University, Olaus Magnus väg, 583 30 Linköping, Sweden; Global Diving Research SL, 46004 Valencia, Spain; Hopkins Marine Station, Stanford University, 120 Ocean View Blvd, Pacific Grove, CA 93950, USA; Marine Mammal Research Program, Hawaiʻi Institute of Marine Biology, University of Hawaiʻi at Mānoa, 46-007 Lilipuna Road, Kaneohe, HI 96744, USA

**Keywords:** Behaviour, anthropogenic disturbance, bioenergetics, biologging, daily energy expenditure, humpback whale, reproduction, UAS-photogrammetry

## Abstract

Quantifying energy expenditure of cetacean mother–calf pairs is essential for modelling population-level responses to environmental change and anthropogenic disturbance, especially for capital breeders where mothers face the compounding demands of fasting while lactating. Technological advances in biologging and unoccupied aerial system (UAS; drone) photogrammetry help overcome challenges in translating respirometry methods from animals under human care to the field, creating better energy-use estimates in large cetaceans. We quantified energy expenditure of humpback whale (*Megaptera novaeangliae*) mothers and calves on their winter breeding ground in Hawaiʻi using 51 biologging suction-cup tag deployments paired with simultaneous UAS-photogrammetric measurements (*n* = 20 mothers, *n* = 31 calves) from 2020 to 2025. We used two independent methods, Thrust Power and Breathing Frequency, to calculate per-second energy expenditure, divided deployments into resting dives and other behavioural states and modelled state-specific energy use and daily energy expenditure for mothers and calves. Calves spent more time in resting dives as they aged, and energy expenditure was on average 44% and 35% lower during resting dives for mothers and calves, respectively, compared to other behaviour. Simulated daily energy expenditure for calves ranged from 92 MJ for the smallest and youngest (3.5 m, 1 day) to 452 MJ for the largest and oldest (7.7 m, 110 days), and for mothers from 817 MJ for small females (10 m, 15 000 kg) to 2013 MJ for large females (14 m, 50 000 kg). To illustrate the conservation implications of these energy savings, we simulated the energetic cost of vessel-disturbance-induced reductions in resting behaviour for a representative mother–calf pair. Under a realistic daytime disturbance scenario, daily pair energy expenditure increased by 64 MJ day^−1^, accumulating to an additional 150 kg of maternal blubber catabolized over 60 days of disturbance. Our findings provide best estimates of humpback whale mother–calf daily and behavioural state-specific energy expenditure, enabling improved quantification of the energetic consequences of disturbance.

## Introduction

Energy flow underpins all biological processes, shaping how organisms survive, reproduce and respond to their environment ( Boyd, 2002; Crossin *et al.*, 2014; Chimienti *et al.*, 2020). The efficiency with which individuals acquire, allocate and expend energy directly influences fitness, particularly during energetically demanding reproductive periods (van Noordwijk & de Jong, 1986). For reproductive females, balancing the costs of self-maintenance and offspring provisioning requires energetic trade-offs that consider current investment and future survival and reproductive probability of both the calf and the mother herself (Hamel *et al.*, 2010; Shuert *et al.*, 2020; McHuron *et al.*, 2023). Methods that quantify energetic supply and demand in reproductive individuals and their offspring are therefore essential for linking behaviour to physiology and population status (Gallagher *et al.*, 2018; Adamczak *et al.*, 2023; van Aswegen *et al.*, 2025b, 2025a). While on their low latitude breeding grounds, lactating female humpback whales exemplify these constraints, supporting the high energetic costs of calf rearing entirely from stored reserves ( Clapham, 2000; van Aswegen *et al.*, 2025a). In this study, we compare two complementary methods to quantify rest-driven energy savings and use the Thrust Power (TP) method to simulate daily energetic expenditure in humpback whale mothers and calves during the breeding season.

Linking energy budgets of reproductive females and offspring to individual health and population dynamics is especially critical for recovering populations facing ecosystem changes and increasing anthropogenic disturbance (McHuron *et al.*, 2023). Population Consequences of Disturbance (PCoD) modelling provides a framework for scaling environmental and disturbance-driven changes from behavioural responses to individual energy allocation, health, demographics and population trends (Pirotta *et al.*, 2018). Within this framework, both ecosystem shifts that alter prey availability and disturbances that modify behaviour can influence the energetic balance of individuals, with potential consequences for survival and reproduction. The Hawaiʻi Distinct Population Segment (HDPS) of North Pacific humpback whales (*Megaptera novaeangliae*) was widely considered to have recovered after the global moratorium on commercial whaling (Bettridge *et al.*, 2015). However, over the past decade, the total abundance of HDPS humpback whales has declined by 34% (Cheeseman *et al.*, 2024), accompanied by an 80% reduction in birth rates (Cartwright *et al.*, 2019; Frankel *et al.*, 2022) and a 10-fold reduction in calf survival (Gabriele *et al.*, 2022). These changes coincided with widespread ecosystem disruption associated with the 2014–2016 Pacific Marine Heatwave (Di Lorenzo & Mantua, 2016), which reduced prey availability on the feeding grounds and constrained the energy reserves females carry into the breeding season. Anthropogenic disturbance on the breeding grounds can compound these effects by accelerating the depletion of limited reserves by eliciting energetically costly behavioural responses, such as increased locomotion or disrupted nursing, reducing time and energy available for calf development. By quantifying the overall energetic demands of lactating females and calves across the breeding season, we can more accurately parameterize PCoD models to link environmental change to maternal energy allocation, calf growth and population-level outcomes.

Quantifying energy expenditure of lactating females and calves during different behavioural states is therefore essential for calculating more accurate total energy requirements during the breeding season. It also translates behavioural changes into physiological and demographic outcomes within the PCoD framework (Pirotta *et al.*, 2021; Pirotta, 2022). On the Hawaiian breeding grounds, lactating females spend a large portion of time resting (Glockner-Ferrari & Ferrari, 1985; Cartwright & Sullivan, 2009), reducing their energetic costs while simultaneously providing critical nursing opportunities (Tackaberry *et al.*, 2020; Ratsimbazafindranahaka *et al.*, 2023). Mother–calf pairs typically rest in shallow coastal waters to avoid harassment by males (Craig *et al.*, 2014), which increases their vulnerability to boat traffic and noise, thus disrupting resting behaviour (Lomac-MacNair *et al.*, 2018). Measuring energy expenditure during resting dives versus other behaviour provides a direct way to quantify the costs of disturbance—metrics often inferred indirectly from behavioural response studies or controlled exposure experiments (Dunlop *et al.*, 2013; Sprogis *et al.*, 2020a; Sprogis *et al.*, 2020b; Currie *et al.*, 2021). These behavioural state-specific energetic measurements more accurately determine total daily energy requirements and allow behavioural disruptions to be converted into metabolic costs. When maternal energy reserves are already limited by reduced prey availability on the feeding grounds, additional disturbance that disrupts resting behaviour on the breeding grounds may further constrain maternal energy budgets, with potential cascading effects on calf growth, reproductive output and population recovery.

Traditional methods for quantifying fine-scale energy expenditure of cetaceans, such as respirometry or doubly-labelled water experiments, are limited in their application to animals under human care, or to small cetaceans that can be captured and released (Fahlman *et al.*, 2016, 2018; Rimbach *et al.*, 2021). Bioenergetic studies have outlined two more indirect techniques that incorporate datalogger and unoccupied aerial system (UAS)-derived data to estimate energy expenditure [i.e. field metabolic rate (FMR)] in large free-swimming cetaceans: the “Breathing Frequency” (BF) method (Krogh, 1934; Blix & Folkow, 1995; Rojano-Doñate *et al.*, 2018; Videsen *et al.*, 2023; Christiansen *et al.*, 2023b; Blawas *et al.*, 2025) and the TP method (Gough *et al.*, 2021, 2025). The BF method involves counting breaths to calculate FMR over a period of hours, a method first proposed by Krogh (1934) and more recently used to create estimates of FMR from breathing rates from a range of species (Rojano-Doñate *et al.*, 2018; Videsen *et al.*, 2023; Christiansen *et al.*, 2023b; Blawas *et al.*, 2025). The TP method combines fine-scale swimming metrics from tag data and morphometrics from UAS-photogrammetry to add the propulsive cost of individual tailbeats to an estimate of basal metabolic rate (BMR). Combining these methods helps clarify their respective assumptions and uncertainties, improving future methodological choices.

In this study, we integrated data from biologging tags and UAS-photogrammetry to quantify behavioural state-specific and daily energy expenditure in humpback whale mothers and calves on the Hawaiian breeding ground. We compared deployment-level energy expenditure and energetic savings from resting dives using the TP and BF methods, then used the TP method to simulate daily energy expenditure for mothers and calves of different sizes and calf ages based on daily behavioural budgets and behaviour-specific energetic costs. By linking behavioural state-specific energy expenditure to daily energetic demand, this study provides empirically derived parameters for PCoD modelling and improves our ability to predict how environmental change and anthropogenic disturbance influence maternal investment, calf development and population recovery in humpback whales.

## Materials and Methods

### Data collection and processing

Our two main data sources for this study were CATS (Customized Animal Tracking Solutions) biologging tags and UAS videos (Table 1). All data were collected under appropriate NOAA NMFS permits (nos 21476 and 27 548) and university IACUC protocols. We deployed CATS tags on humpback whale mother–calf pairs each February from 2020 to 2025 from a small boat (<10 m in length) using a 6-m carbon fibre pole following procedures from Friedlaender *et al.* (2009) and Wiley *et al.* (2011). We tagged lactating females (*n* = 27) and calves (*n* = 47). CATS tags were deployed on whales off the coast of Lahaina, Maui, in 2020–2023 and 2025, and along the northwest coast of Hawaiʻi Island in 2024. CATS tags included a suite of sensors to measure orientation and movement: a tri-axial accelerometer, a tri-axial magnetometer, a tri-axial gyroscope, a pressure sensor, a light sensor and a temperature sensor (Cade *et al.*, 2021). The sampling rates were set at 400 Hz for the accelerometers, 50 Hz for the magnetometers and gyroscopes and 10 Hz for the pressure, light and temperature sensors (Cade *et al.*, 2018). The high accelerometer sampling rate allows estimation of swim speed from high-frequency vibrations (“jiggles”) of the tag caused by turbulent water flow, which increase exponentially with swimming speed (Cade *et al.*, 2018). Accelerometer data were retained at 400 Hz to estimate swim speed, after which all sensors were decimated down to 10 Hz for subsequent behavioural and energetic analyses (Cade *et al.*, 2021). CATS tags also included a camera set at 2k or 4k resolution, and two tags included LED headlights triggered by low-light conditions, although these data were not analysed for this study. We also captured high-resolution UAS videos of each tagged whale when weather and light conditions allowed, following procedures from Christiansen *et al.* (2018). We used a DJI Inspire 2 quadcopter with either a Zenmuse X5s or X7 camera to record high-resolution video (3840 × 2160), and a LightWare SF11/C laser altimeter to record altitude (for details, see van Aswegen *et al.*, 2025b).

**Table 1:** Summary of morphometric and CATS tag deployment characteristics of humpback whale mothers (*n* = 20) and calves (*n* = 31) tagged in Hawai’i each February from 2020 to 2025

	Mothers (*n* = 20)	Calves (*n* = 31)
Total length (m) (mean ± SD) (range)	12.46 $\pm$ 0.98 (10.23–14.06)	5.32 $\pm$ 0.52 (4.36–6.41)
Mass (kg) (mean ± SD) (range)	29 669 $\pm$ 6968 (17 154–44 218)	2410 $\pm$ 776 (1246–4454)
Deployment duration (hh:mm) (mean ± SD) (range)	5:56 $\pm$ 3:22 (0:49–10:57)	4:58 $\pm$ 3:47 (0:46–18:04)
Age (days since birth) (mean ± SD) (range)	N/A	36 $\pm$ 20 (6–100)
Total deployment respiration rate (breaths min^−1^) (mean ± SD)	0.59 $\pm$ 0.25	1.77 $\pm$ 0.45
Rest respiration rate (breaths min^−1^) (mean ± SD)	0.39 $\pm$ 0.19	1.24 $\pm$ 0.29
Other behaviour respiration rate (breaths min^−1^) (mean ± SD)	0.74 $\pm$ 0.28	1.87 $\pm$ 0.41
Estimated total lung capacity (TLC_est_) (L) (mean ± SD) (range)	1789 $\pm$ 379 (1061–2609)	173 $\pm$ 51 (89–307)
Estimated tidal volume (V_T_est) (L) (mean ± SD) (range)	912 $\pm$ 193 (541–1331)	88 $\pm$ 26 (45–156)
Fluke planar area (m^2^) (mean ± SD)	3.39 $\pm$ 0.57	0.87 $\pm$ 0.22
Chord length (m) (mean ± SD)	0.97 $\pm$ 0.14	0.55 $\pm$ 0.09

All values are reported as mean ± standard deviation with value ranges where applicable.

Both MATLAB R2024a and R v4.4.2 were used for data analysis. MATLAB was used for initial CATS tag sensor processing, dive detection, behavioural state partitioning, tailbeat detection, TP analysis and Monte Carlo simulation, breath detection and BF analysis and Monte Carlo simulation. R was used for modelling and statistical testing, including TP and BF methods comparison, linear mixed-effects on behavioural state-specific energy expenditure, beta regression models on proportion of time resting, simulation of daily energy expenditure and visualization of results.

### UAS morphometric analysis

First, we selected nadir images from UAS-derived videos for each tagged animal where the animal was in a straight orientation at the surface with the body visible from the head to the tail flukes. Morphometric analysis was conducted using the software Whalength and ImageJ 1.5i (Schindelin *et al.*, 2012; Dawson *et al.*, 2017). We followed methods described by Gough *et al.* (2025) to measure the total body length (TL; m), the chord length (*L*_chord_; m), the linear distance from the notch between the flukes to the anterior insertion of the flukes on the tail, and the fluke planar area (*A*_fluke_; m^2^), a polygonal outline of the flukes.

We estimated body mass for all tagged whales (mothers and calves) using a tissue-composition approach adapted from van Aswegen *et al.* (2025c). For each individual, we first measured total length (TL) and body width (BW) in 5% increments and used methods from Christiansen *et al.* (2019) and van Aswegen *et al.* (2025b) to estimate body volume (BV). Body condition (BC) for each tagged whale was also estimated using methods from Christiansen *et al.* (2019) and van Aswegen *et al.* (2025b) as residuals calculated from the population-wide age-specific relationship between BV and BL, where positive BC values indicate above-average condition and negative values indicate below-average condition for whales of a given body length (van Aswegen *et al.*, 2025b). BV was then partitioned into skeletal muscle, bone and visceral tissues using Equations (11)–(13) from van Aswegen *et al.* (2025c). Blood volume was fixed at 6% of BV (Lockyer, 1976). Blubber volume was estimated from the remaining BV after accounting for structural tissues and blood, adjusted for each whale’s estimated BC (van Aswegen *et al.*, 2025c).

Tissue masses were calculated using tissue-specific densities derived from post-mortem humpback whale calves reported in van Aswegen *et al.* (2025c) (muscle = 972.6 kg m^−3^; bone = 1123.5 kg m^−3^; viscera = 953.7 kg m^−3^; blubber = 985.3 kg m^−3^) and blood densities across mammal species (1048.7 kg m^−3^, IT’IS Foundation 2022). Total body mass was obtained by summing muscle, bone, viscera, blood and blubber masses. This procedure directly applies Equations (9)–(13) of van Aswegen *et al.* (2025c) to each whale’s measured TL and estimated BC, providing individual mass estimates for each tagged animal.

### Energy expenditure—TP method

We followed methods described by Gough *et al.* (2025) to estimate energy expenditure using two independent methods: the TP method and the BF method. The TP method relies on combining the morphometrics of each animal with swimming kinematics to estimate the energetic cost needed to propel a body of a given mass through seawater of a given density at a given speed. We first identified each tailbeat and calculated its oscillatory frequency using custom scripts written in MATLAB R2024a (updated from Gough *et al.*, 2019, 2021, 2025). Following methods from Gough *et al.* (2025), we calculated the thrust power output (${P}_T$; expressed in joules per second, J s^−1^) averaged over each tailbeat period using Equation (1).


(2) \begin{equation*} {P}_{\mathrm{T}}=0.5\rho{C}_{\mathrm{T}}{U}^3{A}_{\mathrm{fluke}}{\left(\frac{h}{L_{\mathrm{chord}}}\right)}^2\kern1em \end{equation*}

where $\rho$ is the density of seawater (1025 kg m^−3^ at 25 C), ${C}_T$ is the coefficient of thrust estimated from experimental hydrodynamic models of lunate-tail swimming (Chopra & Kambe, 1977), $U$ is the mean forward speed of the animal during that tailbeat period (m s^−1^), A_fluke_ is the fluke planar area, *h* is the heave amplitude (m) set at 1/10th of body length (Bainbridge, 1958; Fish, 1998; Gough *et al.*, 2019) and *L*_chord_ is the chord length of the fluke. The coefficient of thrust (${C}_{\mathrm{T}}$) is derived from oscillating hydrofoil experiments and represents the net thrust generated by a flapping propulsor under given kinematic conditions; during steady swimming, this thrust is assumed to balance whole-body hydrodynamic drag, such that the estimated thrust power represents the mechanical power required to overcome drag at the observed swimming speed (Chopra & Kambe, 1977; Gough *et al.*, 2025).

We then converted the thrust power into metabolic power (${P}_{met}$) using values between 0.1 and 0.4 for the aerobic efficiency (${\mu}_{\mathrm{met}}$) (Fish, 1996; Potvin *et al.*, 2021; Gough *et al.*, 2025) and propulsive efficiency $\left({\mu}_{prop}\right)$ estimated from those same models of lunate-tail swimming (Chopra & Kambe, 1977).


(3) \begin{equation*} {P}_{\mathrm{met}}=\frac{P_{\mathrm{T}}}{\mu_{\mathrm{met}}{\mu}_{\mathrm{prop}}}\kern1em \end{equation*}

After calculating the energy cost (${P}_{\mathrm{met}}$) of each tailbeat, we added energy expended from the estimated BMR (Kleiber, 1975) and, for calves, the heat increment of feeding (HIF) to calculate the total amount of energy expenditure over a given time period. To incorporate uncertainty into estimating overall energy expenditure, we conducted a Monte Carlo simulation following Gough *et al.* (2025). For each iteration (*n* = 100), we selected random values for μ_met_ between 0.1 and 0.4, BMR values varying ±10% around 3.39 * M_body_^0.75^ and HIF values varying ±10% around 0.15 * BMR (for calves only) (Supplementary Table S1) (Rechsteiner *et al.*, 2013; Koliopoulou *et al.*, 2025). Because lactating females fast while in Hawaiʻi, we did not include HIF in their energy expenditure calculations.

### Energy expenditure—BF method

For our second energy expenditure method, we used the BF method that relies on respiration rates, body mass and assumptions about tidal volume and oxygen extraction to calculate the metabolic rate (Videsen *et al.*, 2023; Christiansen *et al.*, 2023a; Blawas *et al.*, 2025). We used custom MATLAB R2022a code from the Github repository “respdetect” (Blawas, 2025) to detect every respiration in a deployment, and calculate the overall respiration rate (${R}_{\mathrm{resp}}$; breaths min^−1^). We used the respiration rate and body mass for each tagged whale to estimate the per-second energy expenditure (J s^−1^) using the lung capacity (L_c_) and tidal volume (V_t_) values from Videsen *et al.* (2023) and a modified equation from Christiansen *et al*. (2023a):


(4) \begin{align*} &\notag\mathrm{Field}\ \mathrm{Metabolic}\ \mathrm{Rate}\\&\quad =\frac{1000\ast \left({V}_t\ast{V}_C\ast \kern0.5em {L}_C\ast \kern0.5em {R}_{\mathrm{resp}}\ast{F}_{\mathrm{O}2}\ast{E}_{\mathrm{O}2}\ast{C}_{\mathrm{O}2}\right)}{60}\kern1em \end{align*}

where ${V}_t$is tidal volume (the volume of air exchanged in a single breath, a proportion of vital capacity), ${V}_C$is vital capacity (the maximum volume of air that can be exchanged in a breath, a proportion of lung capacity), ${L}_C$is total lung capacity (L), ${R}_{\mathrm{resp}}$ is respiration rate (breaths min^−1^), ${F}_{\mathrm{O}2}$ is the fraction of oxygen in inspired air, ${E}_{\mathrm{O}2}$ is the oxygen extraction efficiency and ${C}_{\mathrm{O}2}$is the calorific equivalent of oxygen (kJ L^−1^). Following Gough *et al.* (2025), we accounted for uncertainty in the BF parameter estimates by implementing a Monte Carlo simulation (100 iterations) with uniformly random sampling (with replacement) from defined ranges (Supplementary Table S1). V_t_ ranged from 0.4 to 0.8 of V_C_ based on Wahrenbrock *et al.*’s 1974 study using respirometry with a captive grey whale calf (Wahrenbrock *et al.*, 1974; Sumich, 2001). V_C_ ranged from 0.8 to 0.9 of L_C_ from studies across marine mammal taxa (Kooyman, 1973; Fahlman *et al.*, 2018), and oxygen extraction efficiency (E_O2_) ranged from 0.30 to 0.40 (Videsen *et al.*, 2023). L_C_ was calculated using the allometric equation 0.135 * (M_body_^0.92^) (Kooyman, 1973; Fahlman *et al.*, 2011), then multiplied by a scaling factor (0.8–1.2) to account for uncertainty surrounding total lung capacity estimates (Isojunno *et al.*, 2018). Respiration rate (R_resp_; breaths·min^−1^) was allowed to vary by ±10% of the observed average respiration rate for each deployment to reflect individual and contextual variability in breathing patterns. F_O2_ was set at 0.2095 and C_O2_ was set at 20.1 kJ l^−1^.

### Behavioural state classification

To determine how behavioural state may affect energy expenditure, we first divided each deployment into two simple behavioural states: resting dives (“resting”) and all other active behaviours (“other”) (Fig. 3). Resting dives are a well-described and frequently observed behavioural state of humpback whale mother–calf pairs on the breeding grounds, characterized by prolonged, low-activity bottom phases which are assumed to facilitate nursing and energy conservation (Lomac-MacNair *et al.*, 2018; Bejder *et al.*, 2019). Because this behaviour represents a physiologically meaningful low-cost state, we isolated it explicitly from the broader spectrum of behaviours. Based on visual audit of deployments, the “other” behavioural category was dominated primarily by low-speed travel, with less frequent deeper dives. Surface resting behaviour was rarely observed in our deployments. Dives were detected using custom scripts in MATLAB R2024a, where depth records were first low-pass filtered using a zero-phase Parks-McClellan FIR filter (order 35; passband: 0.09 Hz, stopband: 0.2 Hz; sampling rate: 10 Hz), which attenuates variability at timescales shorter than approximately 11 seconds and preserves the broad morphological structure of each dive. Dives were detected as periods when filtered depth exceeded a surface threshold, with events shorter than 2.5 seconds removed. Within each dive, local maxima in the filtered depth profile, defined as samples exceeding both adjacent values, were identified and filtered to retain only those exceeding 75% of the mean depth across all local maxima within that dive, removing minor shallow inflections. The descent phase extended from dive onset to the first retained local maximum, the ascent phase from the last retained local maximum to dive end and the bottom phase comprised the interval between the first and last retained maxima. Unsupervised k-means cluster analysis was used to roughly separate resting dives from other behaviours based on bottom-phase length, maximum depth and bottom-phase speed variance, revealing a distinct group of dives with maximum depths consistently near ~25 m, longer bottom phases and low speed variance (Supplementary Fig. S2). Cluster analysis results were applied to each deployment, and dive profiles were visually inspected to refine resting dive criteria. A dive was classified as resting when its maximum depth was 5–35 m, bottom-phase speed variance was <0.002 m^2^ s^−2^ and the bottom phase lasted >60 s for mothers or >30 s for calves, to account for different dive durations in calves and mothers (Fig. 3; Supplementary Fig. S2). These parameters were not intended to represent strict biological thresholds, but rather operational criteria based on the clustering of dive characteristics (depth, bottom-phase duration and speed variance; Supplementary Fig. S2) and visual inspection of dive profiles, which together captured the majority of resting dives in our dataset.

### Comparison of TP and BF methods

These two methods (BF and TP) produced different estimates of average per-second energy expenditure for each individual deployment. For mothers, we used a paired *t*-test (R v4.4.2) to compare the deployment-level mean energy expenditure (J s^−1^) between the TP and BF methods. Agreement between methods was further evaluated using a Bland–Altman plot to visualize the mean bias and limits of agreement between methods, the concordance correlation coefficient (CCC) to quantify precision and accuracy and a Deming regression that accounted for measurement error in both variables without assuming either method as the true reference.

For calves, the TP method did not explicitly include the metabolic cost of growth, which represents a substantial portion of energy use in humpback whale calves (van Aswegen *et al.*, 2025c). To enable a more equitable comparison with the BF method, we added estimated per-second metabolic growth costs (excluding energy stored in tissue) derived from the linear calf growth model to create the TP + growth method (van Aswegen *et al.*, 2025c). These models incorporate tissue-specific lipid and protein concentrations from seven post-mortem calves and synthesis inefficiencies of 1.251 and 2.102 MJ MJ^−1^, respectively (van Aswegen *et al.*, 2025c) representing the metabolic energy required to synthesize and deposit tissue relative to the energy ultimately stored in those tissues. Because three estimates of energy expenditure were available for calves (BF, TP and TP + growth), we used a repeated-measures ANOVA with Bonferroni-corrected pairwise comparisons (R v4.4.2) to test for differences in deployment-level energy expenditure (J s^−1^) among methods. Because the ANOVA revealed significant differences between methods, further agreement analyses were not pursued, as the methods were not sufficiently interchangeable to warrant characterizing the limits of agreement.

### Resting dive energy savings

To quantify the energetic savings from resting dives, we estimated the per-second metabolic rate (J s^−1^) during resting dives versus all other behaviours using both the TP and BF methods. For the TP method, energy expenditure during each dive was computed as the sum of BMR and the metabolic cost of propulsion during fluking periods, estimated from tailbeat-level thrust power (${P}_{\mathrm{met}}$) as described above. For the BF method, we partitioned total deployment respirations into breaths associated with resting dives versus breaths associated with all remaining behaviours. Resting respiration rate was calculated as the breaths occurring during the surface interval after a resting dive and before the next dive exceeding 3-m depth, divided by the duration of the preceding dive plus its subsequent surface interval. The respiration rate for all other behaviours was computed from the remaining breaths and remaining deployment duration. We used the steady-state assumptions of tidal volume and oxygen extraction to calculate the BF metabolic absolute rate (J s^−1^) and mass-specific rate (J s^−1^ kg^−1^) for each deployment during resting dives and all other behaviour (Equation (4); Supplementary Fig. S3).

Having already assessed whether deployment-level energetic estimates differed between methods, we next evaluated whether both methods produced similar relative energy savings from resting dives. We ran separate linear mixed-effects models for mothers and calves, with per-second metabolic rate (J s^−1^) as the response variable and behaviour (resting dive vs other), method (TP vs BF) and their interaction as fixed effects, and whale ID as a random intercept. A significant Behaviour × Method interaction would indicate that the two methods differ in the magnitude of estimated relative resting-related energy savings, whereas a non-significant interaction would indicate that both approaches yield consistent estimates of relative energetic benefit during resting dives.

### Effect of behavioural state on TP energy expenditure

Although both the TP and BF methods were used to estimate and compare deployment-level energy expenditure and relative resting-dive energetic savings, we used the TP method exclusively for subsequent modelling of daily energy expenditure. This choice was made a priori because the TP method directly links energetic expenditure to swimming kinematics and tailbeat activity, allowing energetic costs to be assigned to specific behavioural states at fine temporal scales. This bottom-up approach also allows separate modelling of other physiological processes, such as growth and digestion, which can then be incorporated explicitly. In contrast, the BF method reflects integrated whole-animal metabolic demand, which combines multiple physiological processes and cannot as readily be attributed to specific behaviours.

To simulate the daily energy expenditure across calves and mothers of all sizes, we first modelled more precise coefficients for the mass-specific amount of energy saved during resting dives. For every individual, we conducted Monte Carlo simulations (*n* = 100 iterations) in which metabolic efficiency, BMR and HIF were randomly varied uniformly within biologically realistic ranges (Supplementary Table S1). This produced a distribution of possible energy expenditure values (J s^−1^) for each behavioural state per whale.

We then divided the energy expended (J s^−1^) in each behavioural state by the Body Mass^0.75^ to scale by the metabolic body size in order to compare energy savings across individuals of different masses (Kleiber, 1975; Oftedal, 2000). Mothers and calves were analysed separately in all subsequent steps.

We fit linear mixed-effects models (package lme4) relating Kleiber-adjusted energy expenditure (J s^−1^ kg^−0.75^) to behavioural state (resting dives vs other), including a random intercept for individual whale ID. For calves, we included a fixed and interaction effect of calf age on resting and other energy expenditure. No model selection or term-dropping procedures (e.g. AIC-based) were applied. Instead, we quantified parameter uncertainty using a bootstrap approach, fitting the same model across our Monte Carlo thrust power results and summarizing the mean, standard deviation and 95% quantiles of fixed-effect estimates across bootstrap replicates. We fit 100 models, one on each iteration from our Monte Carlo simulation results. We determined what percentage of models showed an effect of resting dives on energy expenditure (J s^−1^ kg^−0.75^), or age for the calf models, below a <0.05 significance level. To be used in our further simulations, model effects had to have a *P* value <0.05 in over 50% of our bootstrapped models.

For both mothers and calves, residual diagnostics for all 100 bootstrap mixed-effects models were assessed using DHARMa simulated residuals. We recorded the percentage showing significant non-uniformity or dispersion and confirmed that excluding failed Kolmogorov–Smirnov test models did not alter results. To quantify how much of the total variation in energy expenditure was attributable to differences between individuals, we calculated the intraclass correlation coefficient (ICC) for each bootstrap replicate model. The ICC represents the proportion of total variance explained by the random effect of whale ID, with higher values indicating that individual differences strongly influence energy expenditure. We averaged the ICC across all 100 bootstraps for mothers and calves to summarize the typical contribution of individual identity to variation in energy use.

### Proportion of time in each behavioural state

We used beta regression models with a logit link function (implemented in *betareg*, R v4.4.2) to model the proportion of time spent in resting dives versus other behaviour as a function of calf age. For these models, calf age was included for both mothers (*n* = 20) and calves (*n* = 31), because mother–calf behaviour is synchronized and the proportion of time spent in each behavioural state is expected to change as calves develop. In contrast, in models of energy expenditure across behavioural states, calf age was included only for calves, as maternal energy expended in resting dives versus other states is less directly affected by calf age. We included deployment length as a weight to account for variable observation durations. Model assumptions were checked by plotting Pearson and quantile residuals, which were randomly scattered around zero, indicating that the linear-in-logit assumption was reasonable and the models fit the data adequately. Our tag deployments were biased towards daytime hours, and we assumed that the relative proportion of resting dives and other behaviour was similar between day and night, allowing us to generalize to a 24-hour period. To evaluate this assumption, we examined the few deployments that extended into nighttime hours (Supplementary Fig. S5), which did not show obvious qualitative differences in the occurrence of resting dives between day and night. However, given the limited number of overnight deployments, this assumption remains uncertain and should be interpreted as a potential limitation of our approach.

### Daily energy expenditure

We simulated values of calves’ daily energy expenditure (MJ) by first randomly drawing combinations of maternal body length (10–14 m) and calf age (1–110 days old) from uniform distributions spanning the observed ranges of tagged individuals. This approach ensured that all combinations of maternal length and calf age were equally represented across the simulation space. Using equations from van Aswegen *et al.* (2025c), we derived estimates of calf mass as a function of age and maternal length, accounting for the influence of maternal length on calf size (van Aswegen *et al.*, 2025a). In each daily simulation, we first calculated the probability of resting dive time based on the age of the calf using the fixed effect from our beta regression model (Table 3). We then calculated the Kleiber-adjusted energy expenditure (J s^−1^ kg^−0.75^) in resting dives and active behaviour, based on the age and mass values, using randomly drawn fixed and interaction effects from a normal distribution using the mean and standard deviation of each effect from our 100 generalized linear models (Table 2). We also incorporated the mean random effect values from our 100 models of behavioural effects on energy expenditure to account for random differences between individuals. We finally converted the Kleiber-adjusted energy expenditure rate (J s^−1^ kg^−0.75^) to megajoules per day using the mass of each calf in the simulation. From repeated simulations (*n* = 1000) of pairs of calf ages and maternal lengths, we calculated the mean, upper 95% confidence interval and lower 5% confidence interval of daily energy expenditure (MJ).

**Table 2:** Generalized linear model results (*n* = 100) on the effects of behavioural state on energy expenditure (mean ± 95% confidence intervals)

	Mother	Calf
Active behaviour (intercept) (J s⁻^1^ kg⁻^0.75^) (mean $\pm$ 95% CI)	8.72 $\pm$ 0.33	8.45 $\pm$ 0.88
Resting effect (mean $\pm$ 95% CI)	−4.45 $\pm$ 0.30	−3.30 $\pm$ 0.64
% of models with significant resting effect	100%	84%
Age effect on intercept (mean $\pm$ 95% CI)	N/A	0.018 $\pm$ 0.017
Age interaction on resting effect (mean $\pm$ 95% CI)	N/A	−0.017 $\pm$ 0.014
% of models with significant age effects	N/A	0%
% of models failing the residual KS test	21%	17%
Mean ICC value	0.002	0.379
Mean marginal *R*^2^	0.48	0.46

Model equation mothers: E (J s⁻^1^ kg⁻^0.75^) ~ Behavioural State + (1 | ID). Model equation calves: E (J s⁻^1^ kg⁻^0.75^) ~ Behavioural State + Age + (1 | ID). One hundred percent of mother models and 84% of calf models showed significant (*P* < 0.05) effects of resting dives on energy expenditure.

We simulated values of mothers’ daily energy expenditure (MJ) by first randomly drawing body masses from uniform distributions spanning the observed range of tagged individuals (15 000–50 000 kg). In each daily simulation, we estimated resting dive probability using the intercept from our beta regression model (Table 3), without an effect of calf age, then calculated energy expenditure (J s^−1^ kg^−0^·^75^) for resting dives and other states using mass and randomly drawn fixed effects from the 100 GLMs (Table 2). We also incorporated the mean random effect values from our 100 models of behavioural effects on energy expenditure to account for random differences between individuals. Finally, we converted energy expenditure (J s^−1^ kg^−0^·^75^) to daily totals (MJ) using each simulated mother’s mass. From 1000 simulations across calf ages and maternal masses, we derived mean, 95% upper and 5% lower confidence intervals for daily energy expenditure.

**Table 3:** Beta regression model results on the effects of calf age on the proportion of time spent in resting dives [model: logit(μ) = β₀ + β₁·CalfAge], weighted by deployment length

Age/sex class	Intercept	Calf age effect	Standard error (Age)	P value
Mother	−0.42	−0.002	0.005	0.59
Calf	−1.21	0.008	0.001	<0.001^***^

^***^ *P* value of less than 0.001.

### Simulated energetic consequences of changes in resting behaviour

To illustrate the consequences of altered resting behaviour on maternal energy budgets, we simulated daily energy expenditure, blubber mass change and prey requirements for a representative mother–calf pair under three behavioural scenarios, using mean body masses reported for Hawaiian humpback whales by van Aswegen *et al.* (2025c) [30 880-kg (13-m) lactating female and a 2310-kg calf]. Scenarios were anchored on an empirical baseline proportion of time spent in resting dives (*P*_rest_ = 0.40) derived from the beta regression model fit to activity budget data from lactating female tag deployments in this study (*n* = 20). We modelled two disturbance scenarios, both applying a 30% reduction in resting time based on behavioural responses during vessel noise from controlled exposure experiments in humpback and short-finned pilot whale mother–calf pairs (Sprogis *et al.*, 2020b; Arranz *et al.*, 2021). In the daytime-only disturbance scenario, resting was reduced by 30% during daylight hours (approximately 11 hours day^−1^ in Hawaiʻi during January–March), when vessel traffic is concentrated (Currie *et al.*, 2021), with resting undisturbed at night, yielding a whole-day *P*_rest_ of 0.34. In the full-day disturbance scenario, the 30% reduction was applied across the entire 24-hour period (*P*_rest_ = 0.28), representing an upper bound on the energetic cost of sustained or repeated disturbance. We note that Sprogis *et al.* (2020b) measured this behavioural response during single vessel exposure events; applying it across daylight hours or a full day is therefore an assumption intended to span a plausible range of disturbance costs rather than predict realistic daily energy expenditure. Daily energy expenditure for the mother and calf under each scenario was estimated using the simulation framework described in the daily energy expenditure section, drawing from the posterior distributions of the behavioural state-specific energy rate models (*n* = 10 000 iterations). Total pair energy expenditure was calculated as the sum of maternal and calf daily energy expenditure.

Because lactating females fast on the breeding grounds while supporting both their own and their calf’s metabolism and movement through blubber catabolism, we converted differences in daily energy expenditure relative to the empirical resting baseline into equivalent maternal blubber mass changes. Blubber energy density was estimated by bootstrap resampling (10 000 iterations) following Christiansen *et al.* (2022) and van Aswegen *et al.* (2025a), drawing lipid concentrations from a Gaussian distribution (62.6% ± 14.8% wet weight) and predicting protein concentrations from the linear relationship between the two variables in baleen whale blubber (Christiansen *et al.*, 2022), with calorific equivalents of 39.54 and 23.64 MJ kg^−1^ wet weight for lipid and protein, respectively (Brody, 1968; Lockyer *et al.*, 1985). This yielded a mean blubber energy density of 26.97 ± 5.11 MJ kg^−1^ (95% CI, 16.94–36.98 MJ kg^−1^). Each energy expenditure draw was divided by the corresponding blubber energy density draw to propagate uncertainty through the blubber mass conversion, with results expressed as a percentage of maternal body mass and reported with 95% credible intervals.

Finally, we converted total pair daily energy expenditure under each scenario into prey mass requirements using prey-specific energy densities adjusted for assimilation efficiency, following van Aswegen *et al.* (2025a). Briefly, van Aswegen *et al.* (2025a) estimated mean energy densities for common humpback whale prey types, including krill (*Thysanoessa* sp. and *Euphausia pacifica*) and Pacific herring (*Clupea pallasi*), by randomly drawing 1000 values from ranges of published energy densities (Vollenweider *et al.*, 2011; Chenoweth *et al.*, 2021; Price *et al.*, 2024) and multiplying by assimilation efficiency coefficients drawn from published ranges for baleen whales (80%–93%; Lockyer, 1981; Nordøy *et al.*, 1993; Mårtensson *et al.*, 1994). We report requirements for both prey types as they represent the primary prey of North Pacific humpback whales on their Alaskan and Southeast Alaskan feeding grounds (Szabo, 2015; Chenoweth *et al.*, 2021) and bracket the range of available prey energy densities. We used prey energy density values adjusted for assimilation efficiency for Pacific herring (6.23 MJ kg^−1^) and krill (3.18 MJ kg^−1^) (van Aswegen *et al.*, 2025a).

## Results

### Data collection and processing

We tagged lactating females (*n* = 27) and calves (*n* = 47). After excluding tag deployments with irreparable tag sensor issues or poor-quality drone images, we were left with 20 lactating female tag deployments and 31 calf tag deployments to include in our final analysis.

### Energy expenditure estimates across methods agree for mothers and diverge for calves

**Figure 1:**
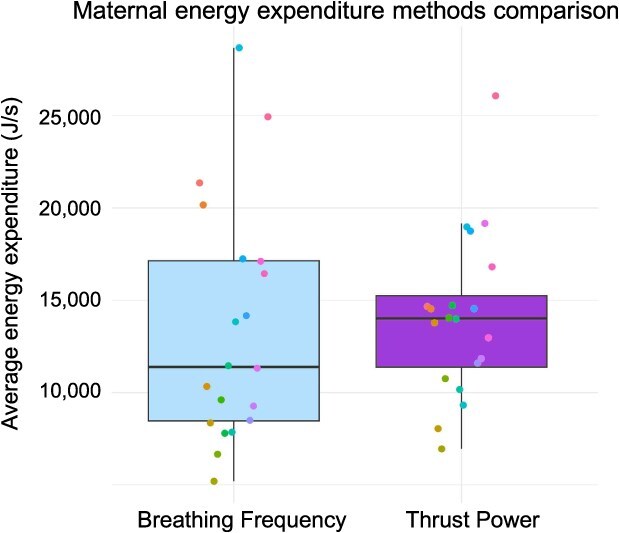
Box-and-whisker plots of maternal energy expenditure in joules per second averaged over 100 bootstrap runs for each deployment (*n* = 20). Each point corresponds to a deployment, coloured by whale ID.

Maternal mean energy expenditure estimates averaged across all deployments did not differ between the BF and TP methods (paired *t*-test, *P* = 0.58). However, comparisons at the individual deployment level revealed systematic differences between the methods. The mean bias between methods was approximately −571 J s^−1^, indicating that TP estimates were slightly higher than BF on average (Fig. 1). Wide limits of agreement indicated substantial variation among individual deployments, with differences between methods varying considerably across whales. The concordance correlation coefficient was 0.66 (95% CI, 0.36–0.83), indicating moderate overall agreement between methods. The Deming regression revealed proportional bias (slope = 1.68), with BF estimates increasing more rapidly than TP at higher energy expenditures. Together, these results indicate that while average differences between methods are small, agreement varies substantially among individuals and diverges systematically at higher energetic levels.

**Figure 2:**
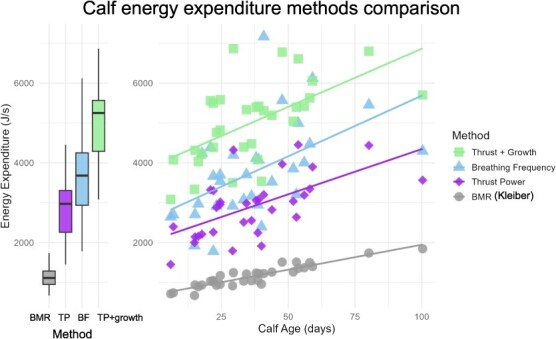
Calf energy expenditure methods comparison between Kleiber BMR (circles), thrust power (diamonds), breathing frequency (blue; triangles) and thrust power plus the metabolic cost of growth (squares). Box-and-whisker plots on the left show energy expenditure in joules per second averaged over 100 bootstrap runs for each deployment. The scatter plot on the right shows the calf age versus energy expenditure estimates based on Kleiber BMR and three different methods, with linear regression lines for clarity. Each calf (*n* = 31) has four points on the scatter plot, corresponding to the four different energy expenditure methods.

For calves, the repeated-measures ANOVA test revealed an effect of estimation method on calf energy expenditure (*F*(1.96, 60.81) = 288.88, *P* < 0.001, η^2^ = 0.725), and post-hoc pairwise comparisons with Bonferroni correction showed that all methods differed significantly from each other (all adjusted *P* < 0.001) (Fig. 2).

### Rest-driven energy savings estimates are consistent across methods

**Figure 3:**
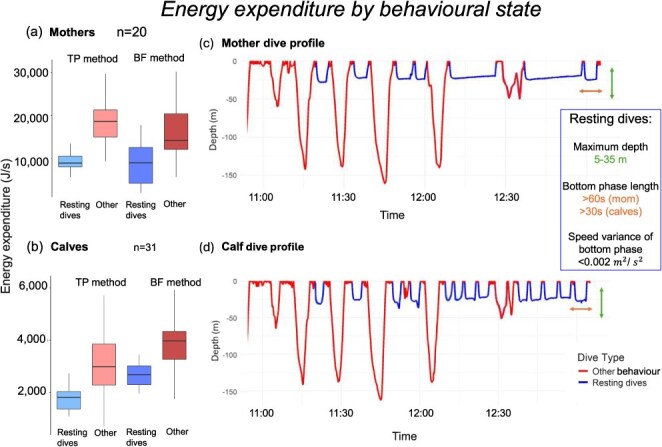
Panels **a** and **b** show the difference in energy expenditure (J s^−1^) between resting dives and other behaviour using the TP and BF methods in mothers (a) and calves (b). Panels c–d show synchronized mother–calf pair dive profile showing resting dives (blue) and all other active behaviour (red). Resting dives are characterized by a maximum depth between 5 and 35 m, a bottom phase longer than 60 seconds for mothers and 30 seconds for calves, a low variance of speed (<0.002 m^2^ s^−2^) during the bottom phase of the dive.

The linear mixed-effects models of energy expenditure as a function of behaviour (resting dives vs other behaviour) and method (TP vs BF), as well as their interaction, were fit for mothers and calves separately. For both mothers and calves, the interaction terms were small and non-significant (mothers: *F*_1,57_ = 0.71, *P* = 0.404; calves: *F*_1,86_ = 0.17, *P* = 0.678), indicating that the TP and BF methods yielded consistent estimates of the proportional reduction in energy expenditure during resting dives. The reduction in energy expenditure associated with resting dives was estimated to be 36% in calves using the TP method, compared to 34% using the BF method. The reduction in energy expenditure associated with resting dives was greater in mothers, but still statistically consistent between methods: 40% using the TP method and 47% using the BF method (Fig. 3).

### Resting dives reduce mass-specific energy expenditure in mothers and calves

All 100 maternal models and 84 of 100 calf models showed a significant effect of behavioural state on metabolic mass-specific energy expenditure, while the interaction between calf age and energy expenditure was not significant in any cases (Table 2).

For mothers, 21% of models showed residuals that significantly deviated from the expected distribution according to the Kolmogorov–Smirnov (KS) test. Excluding these models did not affect results: the reduction in energy expenditure during resting dives was −4.45 J s^−1^ kg^-0.75^ across all models and − 4.47 J s^−1^ kg^-0.75^ after exclusion (<0.5% difference). For calves, 20% of models produced significant KS test results, and excluding these models also did not greatly affect the model results: the reduction in energy expenditure during resting dives was −3.30 J s^−1^ kg^-0.75^ across all models and − 3.29 J s^−1^ kg^-0.75^ after exclusion (<0.5% difference). Given the consistency of the estimates, we retained the original model specification for all subsequent analyses.

### Older calves spent more time in resting dives

The beta regression model indicated that age was positively associated with the proportion of time spent in resting dives for calves (effect = 0.008; *P* < 0.001). This trend should be interpreted with caution, as the effect size was small and variability among individuals was high. The model provided a better fit than an intercept-only model (ΔAIC = 16). In contrast, calf age did not have a significant effect on the proportion of time spent in resting dives for mothers (effect = −0.002; *P* = 0.59), nor did it improve model fit (ΔAIC = −1.7), indicating that maternal resting behaviour did not vary systematically with calf age (Table 3; Supplementary Fig. S1). Residual diagnostics indicated no deviation from normality.

### Daily energy expenditure estimates

**Figure 4:**
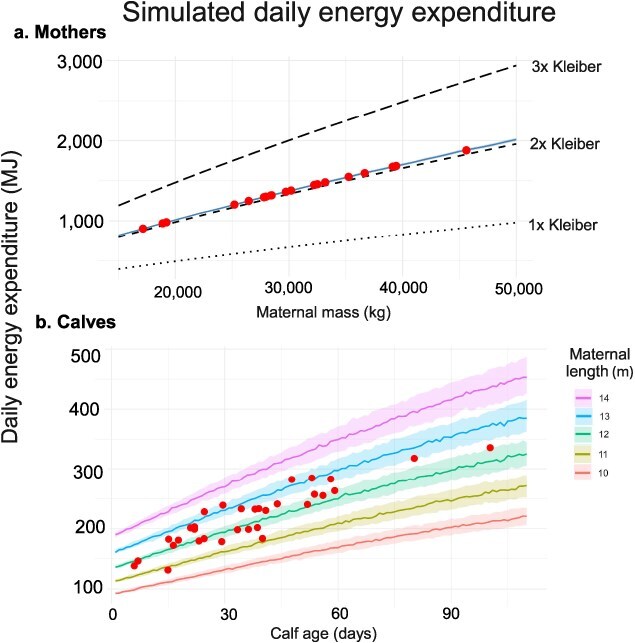
Simulated daily energy expenditure in mothers (**a**) and calves (**b**). The dots on both subplots are the predicted daily MJ energy expenditure of the tagged mothers or calves. For mothers (a), the mean predicted daily MJ is the solid line, with the dotted, dashed and long dashed lines representing the daily energy expenditure at predicted metabolic rates of 1×, 2× and 3× Kleiber, respectively. For calves (b), solid lines show the mean daily energy expenditure of calves born to mothers of different lengths (10–14 m), with 95% confidence intervals shaded.

Simulations predicted that the smallest mothers (15 000 kg) expended 817 ± 8.9 MJ day^−1^. The heaviest mothers (50 000 kg) expended 2013 ± 21 MJ day^−1^ (Fig. 4a). Simulations predicted that the youngest calves born to the smallest mothers (age = 1 day, calf length = 3.53 m, calf mass = 720 kg, maternal length = 10 m) expended 92 MJ day^−1^, while young calves born to larger mothers (age = 1 day, calf length = 4.94, calf mass = 1881 kg, maternal length = 14 m) expended 189 MJ day^−1^ (Fig. 4b). The simulated max energy expenditure for calves was predicted to be 452 MJ day^−1^ (age = 110 days, calf length = 7.74 m, calf mass = 6782 kg, maternal length = 14 m) (Fig. 4b).

### Decreased time in resting dives increases maternal body mass loss and prey requirements

To illustrate how reductions in resting behaviour scale to energetic costs and prey requirements, we simulated daily energy expenditure for a representative mother–calf pair using mean body masses (mother: 30 880 kg, 13 m, calf: 2310 kg; van Aswegen *et al.*, 2025c) under three scenarios anchored on an empirical baseline of 40% of the day spent in resting dives. In the resting baseline scenario (*P*_rest_ = 0.4), total metabolism and movement costs for the representative pair were 1602.8 MJ day^−1^ (95% CI, 1449.7–1755.3 MJ day^−1^), equivalent to 61.8 kg of maternal blubber day^−1^ (95% CI, 42.6–95.1 kg day^−1^), requiring 257.3 kg herring (95% CI, 232.7–281.7 kg) or 504.0 kg krill (95% CI, 455.9–552.0 kg) to meet that demand on the feeding grounds. Under the daytime-only disturbance scenario (*P*_rest_ = 0.34), daily pair energy expenditure increased by 64.1 MJ day^−1^ (95% CI, −139.3 to 264.6 MJ day^−1^), equivalent to an additional 2.5 kg of maternal blubber catabolized per day (95% CI, −5.4 to 10.9 kg day^−1^), or 0.008% of maternal body mass lost per day (95% CI, −0.017% to 0.035%), requiring an additional 10.3 kg herring (95% CI, −22.4 to 42.5 kg) or 20.1 kg krill (95% CI, −43.8 to 83.2 kg day) consumed on the feeding grounds. Under the full-day disturbance scenario (*P*_rest_ = 0.28), daily pair energy expenditure increased by 138.6 MJ day^−1^ (95% CI, −64.6 to 342.3 MJ day^−1^), equivalent to an additional 5.3 kg of maternal blubber catabolized per day (95% CI, −2.4 to 14.6 kg day^−1^), or 0.017% of maternal body mass lost per day (95% CI, −0.008% to 0.047%), requiring an additional 22.2 kg herring (95% CI, −10.4 to 54.9 kg) or 43.6 kg krill (95% CI, −20.3 to 107.6 kg) on the feeding grounds to offset each day of reduced resting (Supplementary Table S2).

## Discussion

In this study, we applied an integrated biologging–UAS framework combining kinematics, behavioural analysis and morphometrics, to estimate fine-scale and daily energy expenditure in humpback whale mothers and calves on their Hawaiian breeding grounds. This approach allowed us to directly compare two methods for estimating FMR in large free-swimming mysticetes—the TP and BF methods—and provide insights relevant to methodological choices for future studies. Across both methods, humpback whale mothers and calves substantially reduced energy expenditure during resting dives, with expenditure rates decreasing by approximately 35%–44% relative to other behaviour. Calves also spent increasing proportions of time resting as they aged, suggesting behavioural shifts that may help offset the energetic costs of rapid growth. Simulations of daily energy expenditure indicated that calves expend approximately 92–452 MJ day^−1^ depending on age and size, while mothers expend 817–2013 MJ day^−1^ while fasting and supporting lactation. These results provide one of the most detailed assessments of energetic demands in a capital-breeding mysticete, directly enabling quantification of the energetic costs associated with disturbance to resting behaviour, and establish essential parameters for future bioenergetic and PCoD models.

### Comparing the TP and BF methods: strengths, limitations and applications

The TP and BF methods offer comparative estimates of energetic expenditure that differ substantially in their assumptions, data requirements and physiological interpretability. The TP method is analytically intensive, requiring high-resolution kinematic data, accurate tailbeat detection and individual morphometrics (i.e. fluke planar area) (Gough *et al.*, 2025). The TP method directly links mechanical work to metabolic cost, resolving energy expenditure at the scale of individual behaviours, and making it well suited for bottom-up bioenergetic models that partition energetics into locomotion, BMR and growth. However, the BMR estimate is based on data from smaller adult terrestrial mammals (Kleiber, 1975), thus not accounting for the potential differences in allometry in marine mammals and growth in calves (He *et al.*, 2023). In contrast, the BF method estimates total metabolic rate from respiration rate and, when properly calibrated, integrates all physiological processes contributing to oxygen demand.

Both methods carry important limitations and uncertainties. As a relatively new approach, the TP method still requires further calibration and validation across species. For the TP method, we assumed that non-fluking periods reflect BMR, and did not model gradual energetic transitions between behavioural states. Whether large cetaceans exhibit elevated BMRs relative to terrestrial mammals remains an open question: some studies suggest higher resting metabolic rates in aquatic mammals (Williams, 2022), whereas others argue that apparent deviations disappear for large animals (He *et al.*, 2023) once methodological biases are corrected (Lavigne *et al.*, 1986), or even that the ¾ Kleiber law is not universal (Darveau *et al.*, 2002; White, 2010). Given this uncertainty, we used Kleiber scaling as a baseline and incorporated ±10% variability in our simulations (see Gough *et al.*, 2025, discussion on model caveats). Notably, resting dive energy expenditure estimated using the BF method in mothers approached Kleiber-predicted values (Supplementary Fig. S3), providing some support for the use of Kleiber scaling as a reasonable baseline for resting metabolic rates in this system. The BF method in this study relies on fixed assumptions about lung capacity, tidal volume and oxygen extraction, and, critically, assumes a steady-state relationship between respiration rate and metabolic rate (Fahlman *et al.*, 2016; Videsen *et al.*, 2023). Respiration-based estimates assume a tight coupling between breathing rate and metabolic demand, but marine mammals can defer oxygen debt and repay it during post-dive recovery, decoupling respiration from instantaneous energy expenditure (Kooyman *et al.*, 1973; Fahlman *et al.*, 2008). Tidal volume and oxygen extraction are known to vary with behaviour and breath rate, and future studies should incorporate the actual dynamics of these parameters to use the BF method more accurately for behavioural energetics (Sumich, 2001; Fahlman *et al.*, 2016; Fahlman *et al.*, 2019).

Our results demonstrate that age class is also an important factor when selecting between the TP and BF approaches. For adult lactating females, deployment-level mean estimates from the two methods were broadly similar, but more studies are needed that pair these approaches across a wider range of sizes and behavioural contexts to determine when their estimates can be considered comparable. In calves, however, the methods diverged: the Simple TP method produced lower energetic estimates than the BF method, whereas the TP + growth method yielded the highest daily expenditures (Fig. 2). These discrepancies highlight the difficulty of estimating metabolic rates in calves that are rapidly growing and developing. The TP method is newer and remains unvalidated against independent measures of energy expenditure, limiting our ability to assess its accuracy across age classes and body sizes. Estimating calf energetics is additionally complicated by high rates of tissue synthesis and changing lipid and protein composition, which introduce uncertainty into growth-related metabolic costs (Adamczak *et al.*, 2023; van Aswegen *et al.*, 2025c). Thus, the estimate of BMR for calves used for the TP method therefore is likely underestimated, and the cost of growth accounted for by the TP + growth method introduces additional uncertainty and assumptions described in van Aswegen *et al.* (2025c). Interestingly, the results for the BF method fell between the two (Fig. 2), but agreement between methods at any given point should be interpreted cautiously, as it may shift substantially depending on the mean parameter values chosen. These caveats underscore the need for continued refinement of both methods and careful consideration of their assumptions when applied across behavioural states or age classes.

### Resting dives reduce energy expenditure during a critical period for mothers and calves

Both analytical approaches yielded similar estimates of reduced energy expenditure during resting dives. In calves, reductions were estimated at 36% using the TP method and 34% using the BF method, whereas in mothers the corresponding estimates were 40% and 47%, respectively (Fig. 3). Comparable reductions in respiration rate during resting behaviour have been observed in other humpback whale populations, for example, lactating females in the gulf of Exmouth had respiration rates half of those of adults on the feeding grounds (Bejder *et al.*, 2019). Quantifying the magnitude of energy savings during rest therefore enables us to estimate the energetic penalties of lost resting time, as observed in several cetaceans exposed to vessel traffic and tourism (Lusseau, 2003; Heenehan *et al.*, 2017; Arranz *et al.*, 2021). Because the TP method also directly links swim speed to energetic cost, it provides a mechanistic framework for translating the small, speed-based behavioural changes commonly measured in disturbance studies into corresponding changes in energy expenditure (Scheidat *et al.*, 2004; Christiansen *et al.*, 2014; Senigaglia *et al.*, 2016; Sprogis *et al.*, 2020a). By using both these kinematic fine-scale and behavioural state-scale perspectives, we can estimate the energetic consequences of altered behaviour, providing a biologically meaningful basis for assessing disturbance impacts on mothers and their rapidly growing calves.

Although the relationship between age and resting dive behaviour did not have a large effect size in our models, incorporating this age-dependent trend into calf daily energy expenditure simulations was biologically reasonable, and preferable to the alternative of assigning all calves the same dive behaviour regardless of age. Multiple studies document age-related changes in humpback calf behaviour across regions: Cartwright and Sullivan (2009) found that young calves in Hawaiʻi spent about 85% of their time travelling (i.e. not resting), decreasing to 47% in older calves, and Ratsimbazafindranahaka *et al.* (2024) similarly found age-related differences in calf behaviour off Madagascar. These patterns suggest that assuming a single behavioural budget across all calf ages would introduce systematic bias into energetic estimates, particularly for the youngest individuals. In terms of absolute resting levels, our predicted resting dive times are consistent with values reported in other studies: Bejder *et al.* (2019) found that calves in Exmouth rested 15%–33% of the time, and Zoidis *et al.* (2014) observed that Hawaiian calves rested more than half the day. Across studies and regions, resting consistently emerges as a key strategy for reducing energetic costs during this energetically constrained period for both mothers and calves.

Several factors likely influenced our estimates of daily resting time. Most tag deployments occurred during daylight hours, requiring us to extrapolate to a full 24-hour period (Supplementary Fig. S4). The available long deployments—one calf tag recorded to 4:00 a.m. and three mothers past midnight—showed no clear diurnal changes in diving or swim speed (Supplementary Fig. S5). Yet visual observations indicate resting may peak early in the morning (Helweg & Herman, 1994), suggesting our estimates could be conservative. We excluded resting at the surface because of its rarity in our dataset and the difficulty of distinguishing it in the accelerometer data due to wave action. As a result, our estimates likely underestimate total resting time, which may be important for applying this approach to populations or locations where surface resting is common. Although our models did not detect an effect of calf age on the proportion of time mothers spent in resting dives, previous work shows strong mother–calf synchrony in resting and diving patterns (Bejder *et al.*, 2019; Huetz *et al.*, 2022). Resting behaviour is also likely shaped by factors we could not measure, such as male escort presence (Cartwright & Sullivan, 2009), vessel activity (Sprogis *et al.*, 2020b), water depth and proximity to shore (Meynecke *et al.*, 2021). Together, these factors introduce uncertainty into our estimates, underscoring the need for future deployments with broader temporal coverage and environmental context.

### Daily energy expenditure estimates vary across mysticete species

Our modelled daily energy expenditure estimates for humpback whale calves (92–452 MJ day^−1^ across ages and sizes) using the TP method represent only the costs of basal metabolism, digestion and locomotion. These components accounted for ~30%–40% of calves’ estimated total daily energy requirements that include somatic growth, tissue synthesis and assimilation efficiency (van Aswegen *et al.*, 2025c). Comparisons of daily energy expenditure with previous estimates for mysticete calves must be interpreted cautiously because methods differ in how they incorporate growth. In our TP framework, we explicitly separate locomotor costs from the metabolic cost of synthesizing new tissue, whereas respiration-based FMR estimates inherently include tissue synthesis costs. As a result, our daily energy expenditure values are not directly equivalent to respiration-derived FMRs, unless tissue synthesis is explicitly added, as in the case of the TP + growth method, or the BF method is scaled up to a full 24-hour estimate, both of which are beyond the scope of this study. With that caveat, our TP method estimates were generally similar to previous respiration-based values for humpback (HBW) calves and grey whale (GW) calves, and were well below values for southern right whale (SRW) calves. Christiansen *et al.* (2025) estimated FMRs of ~200–300 MJ day^−1^ for 4- to 6-m humpback calves in Western Australia, similar to our TP-based estimates. Sumich (2021) reported FMRs of 125 ± 25–330 ± 49 MJ day^−1^ for grey whale calves between 4.7 and 6.7 m in length, also within the range of our daily energy estimates. Our tagged calves fell within a similar length range to the grey whale calves, but weighed significantly more, reflecting species differences in size and growth rates (HBW: 4.4–6.4 m and 1246–4454 kg versus GW: 4.7–6.7 m and 1158–3539 kg). SRW calves exhibited much higher FMRs (730 ± 230 MJ day^−1^;  Nielsen *et al.*, 2019), consistent with their substantially larger body masses (SRW mean: 3880 kg versus HBW mean: 2410 kg). Although the BF and TP methods are not directly comparable for calves, cross-species comparisons help contextualize energetic patterns among capital-breeding mysticetes.

Our modelled daily energy expenditure estimates for humpback mothers (817–2013 MJ day^−1^) using the TP method are more directly comparable to previous studies, as adults do not have significant growth costs. Maternal basal metabolism and locomotion costs were between 48% and 53% of their total daily energy loss while on the breeding ground, a significant cost for a lactating female (van Aswegen *et al.*, 2025a). Our estimates had a larger range than those modelled for adult humpbacks on the Australian breeding ground (~700–800 MJ day^−1^) (Christiansen *et al.*, 2025). Although both studies estimated the cost of basal metabolism and locomotion in HBW lactating females, Christiansen *et al.* (2025) used respiration rates when whales were at rest and the BF method to calculate basal metabolic costs, and added locomotor costs based on a regression between respiration rate and horizontal swim speed. Our maternal energy estimates are approximately half of those reported by Videsen *et al.* (2023) for adult humpbacks on the feeding grounds (~1500–3500 MJ day^−1^). This difference is expected, as whales actively foraging and digesting prey experience substantially higher energetic costs than fasting females on the breeding grounds (Booth *et al.*, 2023). Reduced energetic expenditure in mothers likely reflects both behavioural and physiological adaptations to fasting, including decreased locomotor activity, prolonged resting bouts (Bejder *et al.*, 2019) and possible metabolic suppression (Crocker & McDonald, 2022). Despite lower overall energy use, a large proportion of the mother’s energy budget is allocated to milk production and calf care, leading to significant depletion of maternal lipid reserves over the breeding season (Christiansen *et al.*, 2016). By subtracting this maternal daily energy expended through metabolism and movement from seasonal UAS-derived energy loss (van Aswegen *et al.*, 2025a), we will be able to estimate the amount of energy transferred to the calf through lactation, a key parameter for bioenergetic models.

### Applications for population consequences of disturbance modelling

Key challenges in PCoD modelling are parameterizing the energetic consequences of behavioural changes and estimating accurate total energy requirements (Keen *et al.*, 2021; McHuron *et al.*, 2022; Noren & Rosen, 2023). Our results address this gap by quantifying both behavioural state-specific energetic costs and total daily energy expenditures for lactating females and calves, providing two complementary inputs for PCoD models. For future behavioural response studies, these state-specific estimates directly link disturbance-induced reductions in resting time to quantifiable increases in metabolic demand, the critical connection between behaviour and individual energy balance.

The biological consequences of increased energy expenditure are most immediately relevant on the Hawaiian breeding grounds, where lactating females fast while supporting both their own metabolism and calf energy requirements through blubber catabolism. To illustrate how disturbance-induced reductions in resting behaviour scale to energetic costs, we simulated energy expenditure for a representative Hawaiian mother–calf pair [30 880-kg (13-m) mother, 2310-kg calf; van Aswegen *et al.*, 2025c] under two disturbance scenarios anchored on an empirical baseline of 40% of the day spent in resting dives. Under the daytime-only disturbance scenario, daily pair energy expenditure increased by 64.1 MJ day^−1^ (3.9% above baseline), equivalent to an additional 2.5 kg of maternal blubber catabolized per day; under the full-day disturbance scenario, this rose to 138.6 MJ day^−1^ (8.5% above baseline) and 5.3 kg of blubber per day. Although credible intervals span zero, reflecting uncertainty in blubber energy composition, these estimates are consistent across scenarios and represent modest increases above baseline daily energy expenditure. The 30% reduction in resting time applied in both scenarios is based on the behavioural response observed in humpback and short-finned pilot whale mother–calf pairs during controlled vessel noise exposure experiments (Sprogis *et al.*, 2020b; Arranz *et al.*, 2021), but those experiments measured acute responses during single vessel encounters. Scaling this response to daylight hours, when whale-watching vessel traffic is concentrated in Hawaiian waters (Currie *et al.*, 2021), represents an approximation of the energetic cost of a day with repeated vessel encounters (*P*_rest_ = 0.34, daytime-only scenario). Although this assumes sustained daytime exposure, near-continuous daytime disturbance has been documented in Hawaiian cetaceans, with spinner dolphins exposed to human activity for 82.7% of daytime hours with a median of only 10 minutes between events (Tyne *et al.*, 2018), suggesting the daytime scenario is not unrealistic for areas of concentrated vessel traffic. Reducing resting behaviour across the full 24-hour day represents a deliberate upper bound that would require near-continuous disturbance (*P*_rest_ = 0.28, full-day scenario). Translating these illustrative scenarios into the actual energetic cost of disturbance to mother–calf pairs would require empirical data on the frequency, duration and spacing of disturbance events, as the cumulative exposure an animal experiences is a key determinant of energetic consequences (Tyne *et al.*, 2018). For a fasting animal that cannot compensate through increased foraging, repeated disturbances across the breeding season accumulate and drain the maternal energy reserves that support calf growth and migration.

Scaling these daily disturbance costs to maternal energy budgets and reproductive success depends on breeding ground residency time and maternal size. With mother–calf resighting intervals of at least 55 days within a season (van Aswegen *et al.*, 2025a; Evans *et al.*, 2026), a pair experiencing daytime-only disturbance across 10, 30 or 60 days would accumulate an additional ~25, ~75 or ~150 kg of maternal blubber loss, respectively (0.08%, 0.24% and 0.49% of maternal body mass), roughly doubling under full-day disturbance. These illustrative simulations used a mother–calf pair of average masses, but the reproductive consequences of disturbance are not uniform across maternal sizes. Body size exerts a strong negative effect on mass-specific energy expenditure in humpback whales, with smaller lactating females losing a proportionally greater fraction of their already lower absolute blubber reserves over the breeding season (Christiansen *et al.*, 2025). For smaller or younger primiparous females, even modest cumulative deficits from repeated disturbance may deplete maternal reserves enough to constrain calf growth and survival (Christiansen *et al.*, 2025; van Aswegen *et al.*, 2025a).

Beyond the breeding season, each day of daytime disturbance on the breeding grounds would require an additional 10.3 kg of herring or 20.1 kg of krill recovered on the feeding grounds; under full day disturbance, this rises to 22.2 kg of herring or 43.6 kg of krill. We report prey requirements for krill and Pacific herring as they bracket the range of prey energy densities available to Hawaiian-wintering humpback whales, where krill represent the primary volumetric component of the diet and Pacific herring the higher-energy alternative (Krieger & Wing, 1986; Witteveen *et al.*, 2011; Szabo, 2015); actual prey mass required to offset a given deficit will therefore vary with local prey composition and availability. While these simulations are not exact predictions of actual disturbance costs, they demonstrate how state-specific energetic estimates can be scaled from individual behaviour to maternal energy budgets and prey requirements, key steps for PCoD modelling. As increasingly frequent marine heatwaves reduce prey availability (Di Lorenzo & Mantua, 2016; Oliver *et al.*, 2018; Oliver *et al.*, 2019), disturbance-driven increases in energetic demand put pressure on already constrained maternal energy budgets, potentially reducing population birth rates and growth rate.

## Conclusions

This study demonstrates the power of combining high-resolution biologging tags with UAS-photogrammetry to quantify fine-scale and daily energy expenditure in large free-swimming whales. By integrating behavioural, kinematic and morphometric data, we provide unprecedented insight into the energetic demands of lactating humpback whales and their calves. These methods offer a framework for expanding energetic studies to other baleen whale species and provide a mechanistic basis for PCoD and bioenergetic models. By combining our high-resolution tag data with our long-term UAS-photogrammetry dataset (van Aswegen *et al.*, 2025c), we can directly link short-term behavioural energetics to seasonal energy budgets, providing a powerful framework for quantifying the value of these protected breeding grounds and informing management strategies.

## Supplementary Material

Web_Material_coag041

## Data Availability

The data that support the findings of this study are available from the corresponding author upon reasonable request.
