# Supplementary material for: Energetic benefits of resting behaviour in humpback whale mother–calf pairs revealed by biologging and UAS-photogrammetry
Source: Conserv Physiol. 2026 Jul 7;14(1):coag041. doi: 10.1093/conphys/coag041 (PMC13339960; doi:10.1093/conphys/coag041)
Supplement: Web_Material_coag041 [file web_material_coag041.zip › Supplementary materials_2.pdf]

Supplemental Materials

Monte Carlo variables	Thrust power method	Breathing Frequency method
Kleiber basal metabolic rate (BMR)	0.9* BMR- 1.10 * BMR	-
Metabolic efficiency (μ_{met})	0.1-0.4	-
Heat increment of feeding (calves only)	0.05*BMR – 0.25*BMR	-
Tidal volume (V_t)	-	0.4-0.8 *V_C
Vital capacity (V_C)	-	0.8-0.9*L_C
Total lung capacity (L_C)	-	0.8-1.2 * L_C
Oxygen extraction coefficient (E_{O2})	-	0.3-0.4
Respiration rate	-	0.9-1.1*RespRate

Table S1: Variable ranges used in the Monte Carlo simulations for the Thrust Power (TP) and Breathing Frequency (BF methods). The simulations used uniformly randomly sampled parameters from the given ranges.

	**Baseline (p_{rest} = 0.40)**	**Daytime disturbance (p_{rest} = 0.34)**	**Full-day disturbance (p_{rest} = 0.28)**
**Daily energy expenditure (MJ day^{-1}), mean (95% CI)**			
Mother-calf pair total	1,635.8 (1,492.5–1,781.0)	1,699.9 (1,559.2–1,841.3)	1,774.4 (1,631.5–1,917.7)
Additional pair DEE relative to baseline	—	64.1 (−93.3–264.6)	138.6 (−64.6–342.3)
**Maternal blubber loss (kg day^{-1}), mean (95% CI)**			
Baseline blubber loss	63.1 (43.6–97.3)	65.6 (45.5–102.0)	68.5 (47.6–105.2)
Additional loss relative to baseline	—	2.5 (−5.4–10.9)	5.3 (−2.4–14.6)
Additional % maternal body mass loss	—	0.008 (−0.017–0.035)	0.017 (−0.008–0.047)
**Prey requirement (kg), mean (95% CI)**			
Baseline- Pacific herring	262.6 (239.6–285.9)	272.8 (250.3–295.5)	284.8 (261.9–307.8)
Baseline- Krill	514.4 (469.3–560.0)	534.5 (490.3–579.0)	558.0 (513.0–603.0)
Additional prey over baseline- Pacific herring	—	10.3 (−22.4–42.5)	22.2 (−10.4–54.9)
Additional prey over baseline- Krill	—	20.1 (−43.8–83.2)	43.6 (−20.3–107.6)

Table S2: Simulated daily energy expenditure, maternal blubber catabolism, and prey requirements for a representative Hawaiian humpback whale mother–calf pair under a baseline and two disturbance scenarios. Maternal and calf body masses were set to mean values reported for Hawaiian humpback whales by van Aswegen et al. (2025c): 30,880 kg and 2,310 kg respectively. The empirical baseline proportion of time spent in resting dives (prest = 0.40) was derived from a beta regression model fitted to activity budget data from biologging tag deployments on lactating females in this study. The daytime-only disturbance scenario applies a 30% reduction in resting time during daylight hours only (~11 hours day^{-1}; prest = 0.34), and the full-day disturbance scenario applies the same reduction across 24 hours (prest = 0.28). Energy expenditure was

estimated by drawing 10,000 values from the posterior distributions of behavioral state-specific energy rate models. Blubber energy density was bootstrapped from baleen whale lipid and protein concentrations following Christiansen et al. (2022) and van Aswegen et al. (2025b), yielding a mean ± SD of 26.97 ± 5.11 MJ kg^{-1}. Prey requirements were estimated using energy densities adjusted for assimilation efficiency following van Aswegen et al. (2025b): 6.23 MJ kg^{-1} for Pacific herring (Clupea pallasi) and 3.18 MJ kg^{-1} for krill (Thysanoessa sp. and Euphausia pacifica). Additional blubber catabolism and prey requirements represent increases relative to the baseline scenario. DEE = daily energy expenditure

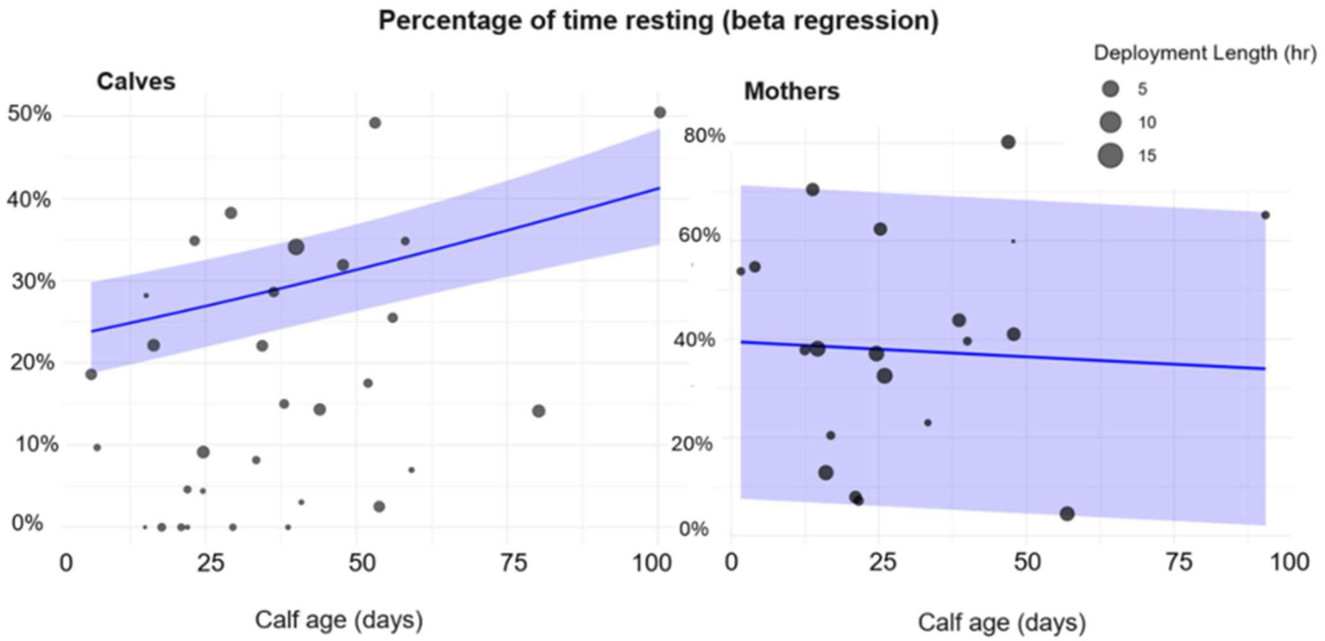

Figure S5: Percentage of time spent resting for mothers and calves as calves age from our beta regression model. Calves n=31, mothers n=20. Dots are sized by total length of the deployment. Blue line is the average model predicted percentage of time resting and shaded 95% confidence intervals

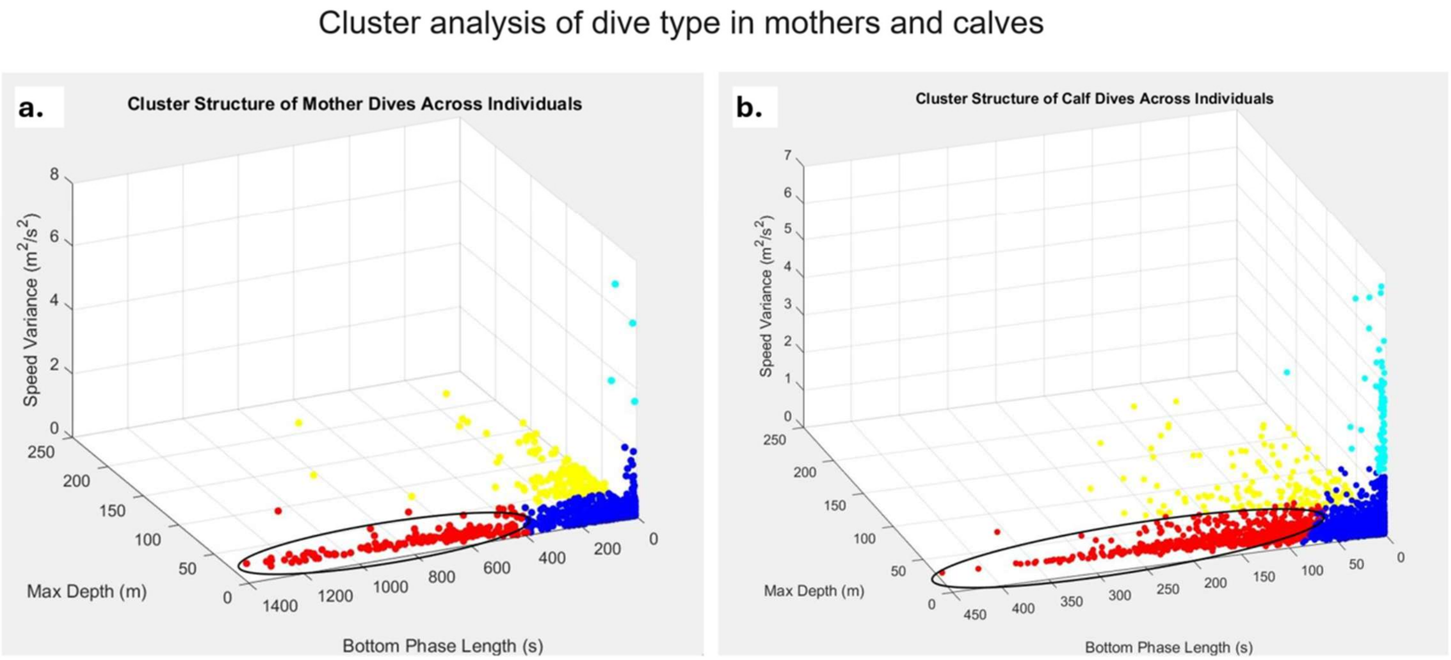

Figure S6: Dives clustered using k-means analysis across bottom phase length (s), maximum depth (m), and speed variance during the bottom phase (m^2s^{-2}) for mothers (a) and calves (b). Mothers n=20 individuals, n=2,347 dives; calves n=31 individuals, n= 5,446 dives. The red cluster roughly represents the resting dives, whose exact parameters we refined by inspecting each individual dive profile and adjusting accordingly.

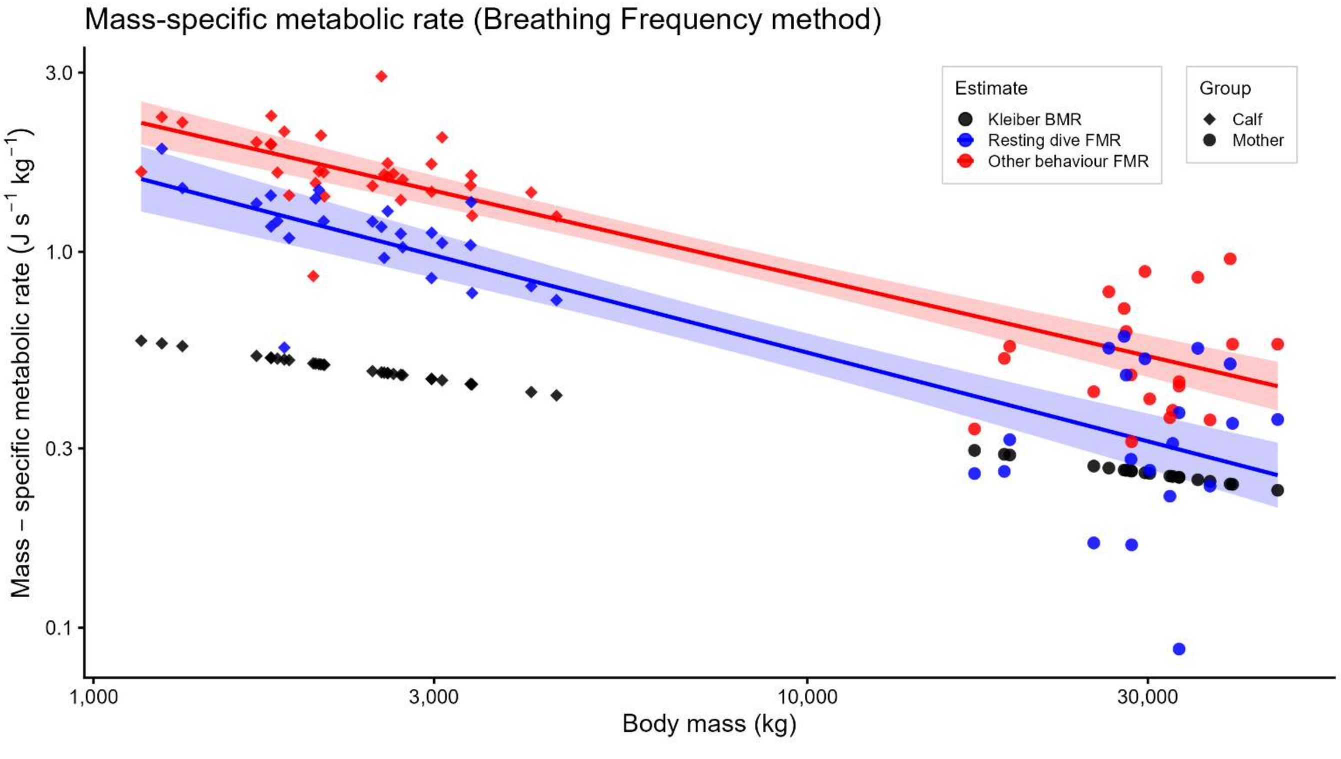

Figure S7: Mass-specific metabolic rate ($J\ s^{-1}\ kg^{-1}$) from the Breathing Frequency (BF) method across calves (diamonds, n = 31) and mothers (circles, n = 20). Points show individual deployment mass-specific metabolic rates estimated using Kleiber BMR (black), resting dive respirations (blue), and all other respirations (red). Log-linear fits with 95% confidence intervals are shown for resting dive FMR (blue): $\log_{10}(MR) = -0.495 \cdot \log_{10}(M) + 1.712$, $R^2 = 0.745$; and other behaviour FMR (red): $\log_{10}(MR) = -0.440 \cdot \log_{10}(M) + 1.693$, $R^2 = 0.797$, where MR is mass-specific metabolic rate ($J\ s^{-1}\ kg^{-1}$) and M is body mass (kg).

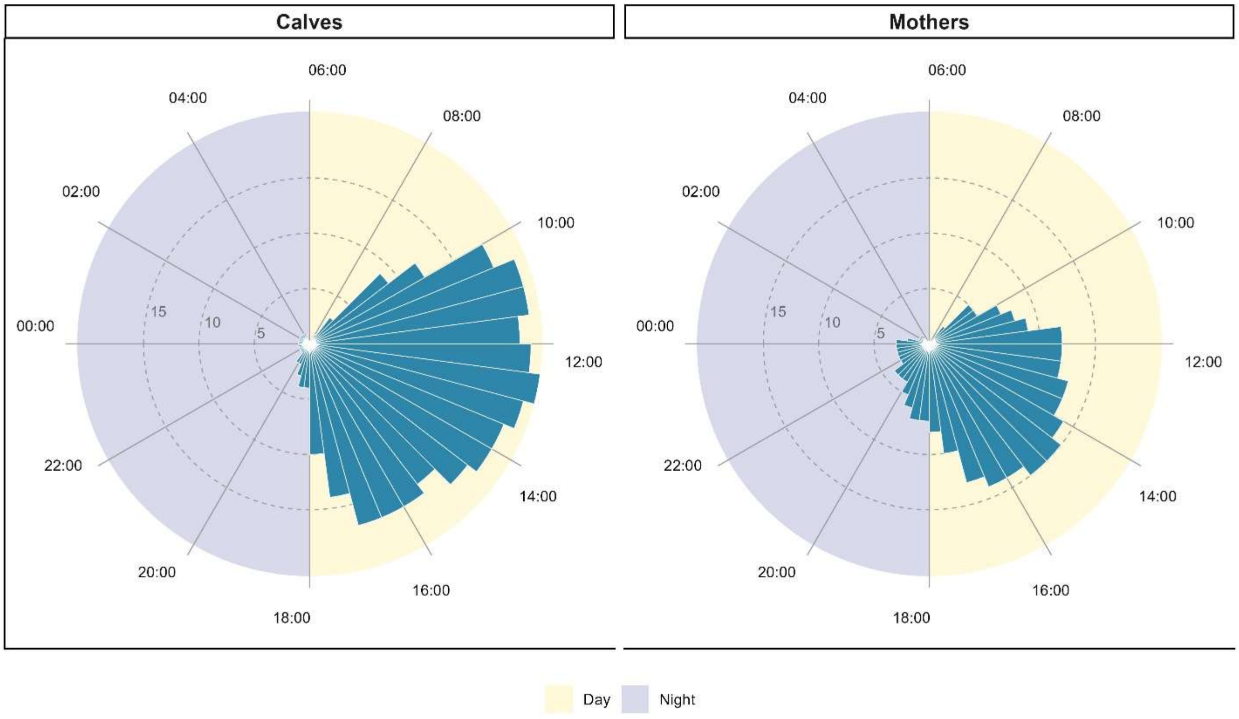

Figure S8: Rose diagram showing 24-hour tag deployment coverage divided into 30-minute bins across calves and mothers. Approximate nighttime hours are shaded in purple and daytime hours shaded in yellow.

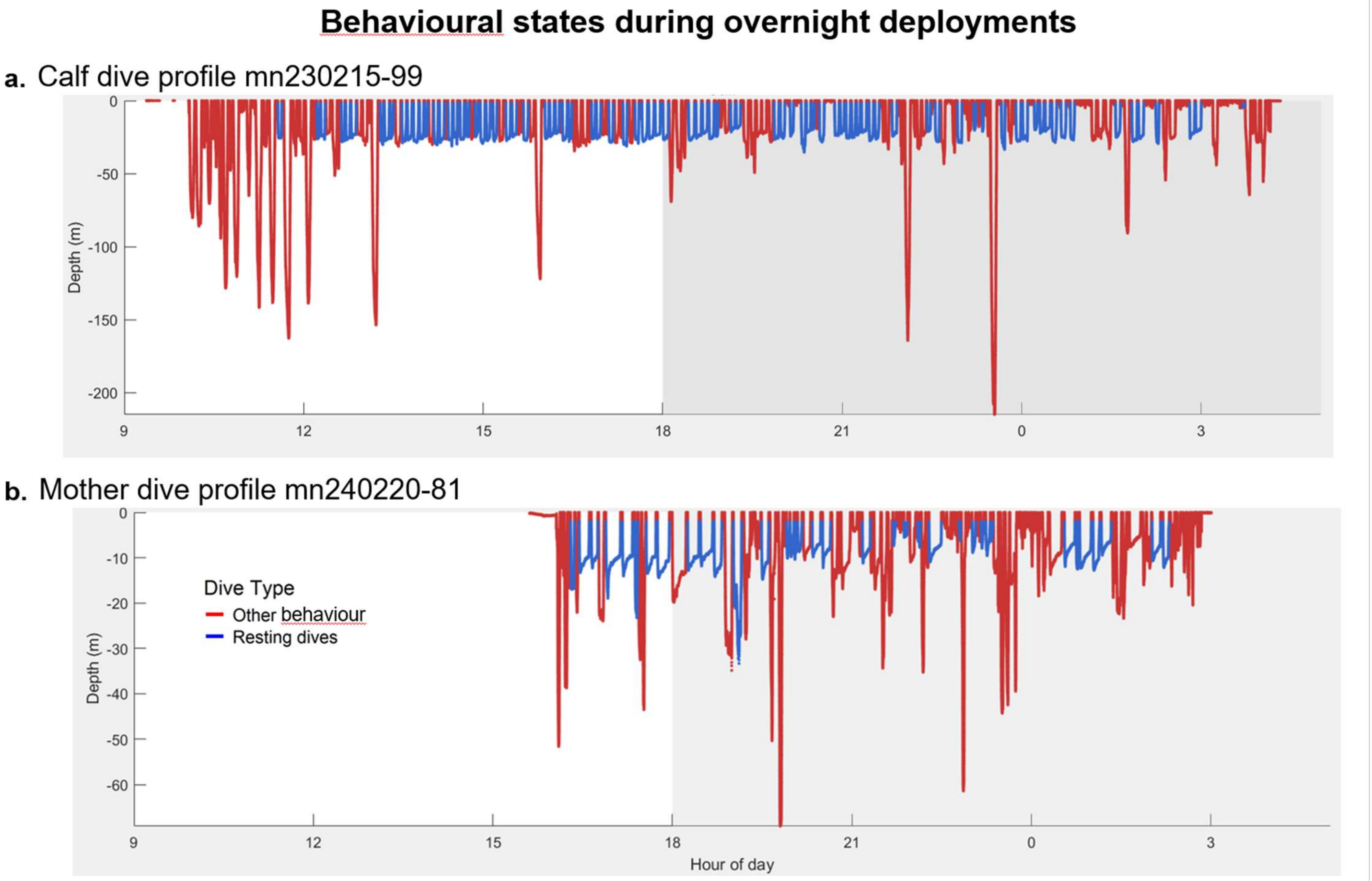

Figure S9: Dive profiles from non-synchronized tag deployments from one calf and one mother that stayed on into the night. Depth is on the y-axis and hour of the day is on the x-axis with approximate nighttime shaded gray. Resting dives are shown in blue, other behaviour is shown in red.
